# ShaoYao decoction ameliorates colitis-associated colorectal cancer by downregulating proinflammatory cytokines and promoting epithelial-mesenchymal transition

**DOI:** 10.1186/1479-5876-12-105

**Published:** 2014-04-26

**Authors:** Xiaochang Lin, Zhiyong Yi, Jianxin Diao, Meng Shao, Liang Zhao, Hongbing Cai, Qin Fan, Xueqing Yao, Xuegang Sun

**Affiliations:** 1 Department of Traditional Chinese Medicine, Nanfang Hospital, Southern Medical University, Guangzhou 510515, China; 2 The Key Laboratory of Molecular Biology, State Administration of Traditional Chinese Medicine; School of Traditional Chinese Medicine, Southern Medical University, Guangzhou 510515, China; 3 Department of Pathology, Nanfang hospital, Southern Medical University, Guangzhou 510515, China; 4 Department of Gastrointestinal Surgery, Guangdong General Hospital, Guangzhou 510120, China

**Keywords:** Shaoyao decoction, Colitis, Colorectal cancer, Epithelial-mesenchymal transition, Snail, Tumor associated macrophages, Proinflammatory cytokines

## Abstract

**Abstracts:**

**Background**

Shaoyao decoction (SYD) is a traditional Chinese medicine prescription formulated by Liu Wan-Su, a master of traditional Chinese medicine in Jin-Yuan Dynasty. SYD is effective in treating ulcerative colitis. Paeonol, a component of SYD, inhibits colorectal cancer (CRC) cell proliferation and induces CRC cell apoptosis. In this study, azoxymethane (AOM)/dextran sodium sulfate (DSS)–induced colitis-associated CRC (caCRC) model and CRC cell lines were used to examine the effects of SYD on CRC in vivo and in vitro.

**Methods**

A translational medicine strategy based on phytomics quality control was adopted. Liquid chromatography was employed for the chemical characterization and chemical fingerprinting of SYD. Protein expression and macrophage existence were determined by immunohistochemistry and western blot. Serum cytokines were quantified by Luminex assay.

**Results**

AOM/DSS-induced caCRC phenotypically resembled human caCRC. SYD significantly increased the survival rate of the mice, ameliorated the general well-being of the mice, and reduced the incidence and multiplicity of colonic neoplasms. SYD inhibited epithelial–mesenchymal transition (EMT), as indicated by upregulated epithelia cadherin and downregulated neuronal cadherin, fibronectin, vimentin, and transcription factor Snail. SYD reduced the expression levels of serum interleukin 1β, interleukin-6, tumor necrosis factor α, tumor-associated macrophages, and p65. These results showed that SYD can attenuate proinflammatory cytokines and inhibit EMT.

**Conclusions**

SYD ameliorates caCRC by suppressing inflammation and inhibiting EMT. SYD might be an alternative therapy for caCRC.

## Introduction

Approximately 142,820 (9%) and 50,830 (9%) new cancer cases and deaths of colorectal cancer (CRC) were reported in the US in 2013, respectively [1]. CRC is the third most common cancer in men and the second in women worldwide [2]. Ulcerative colitis, familial adenomatous polyposis, and hereditary nonpolyposis colon cancer syndrome are the three highest risk groups for developing CRC. In a meta­analysis based on 116 studies, the overall prevalence of colorectal cancer in patients with ulcerative colitis was 3.7%. The risk of colorectal cancer increases by 8% in 20 years and by 18% in 30 years [3]. The pathogenesis of colorectal cancer in patients with ulcerative colitis is triggered by inflammation-dependent mechanisms [4]. Furthermore, ameliorating inflammation can control inflammation-mediated reactive oxygen species, microsatellite instability, telomere shortening, and chromosomal instability [4].

Shaoyao decoction (SYD) is a canonical Chinese medicine prescription formulated by Liu Wan-Su in “Plain Questions - anthology on proper therapy for Qi disorder and pathogenesis to save life” in Jin-Yuan dynasty (AD 1186). SYD is commonly used to treat various inflammatory bowel diseases by clearing heat and damp, removing stasis and toxin in the intestine [5]. SYD is effective in ameliorating the major manifestations of ulcerative colitis, such as hematochezia, abdominal pain, diarrhea, and mucosal defense impairment [6,7]. SYD enhances mucous cell proliferation, decreases exfoliated mucosal cell proliferation, and prevents lamina propria edema and neutrophil infiltration [8]. SYD also exerts anti-inflammatory effects that can decrease the expression of intercellular adhesion molecule 1 (ICAM-1) and tumor necrosis factor α (TNF-α) and increase the expression of interleukin (IL)-10 in colon tissues [9]. Paeonol, the primary phenolic component in Radix Paeoniae Alba (Shaoyao), reduces HT29 cell proliferation by downregulating cyclooxygenase-2 and upregulating p27 [10]. Paeonol induces LoVo cell apoptosis by increasing intracellular calcium concentration [11]. However, whether or not SYD can prevent colitis-associated CRC (caCRC) remains elusive.

The epithelial–mesenchymal transition (EMT) serves important functions in the formation of body plan and the differentiation of multiple tissues [12]. EMT is a process in which epithelial cells trans-differentiate and acquire invasive mesenchymal phenotype. Clinical analysis [13] and animal experiments [14] demonstrated that EMT contributes to the loss of intestinal epithelial cells; thus, EMT is recognized as a major contributor to the pathogenesis of inflammatory bowel diseases. Transforming growth factor β (TGF-β), TNF-α, and nuclear factor κB (NF-κB) participate in inflammation development; these factors are also crucial for the initiation of EMT [15]. In the present study, the functions of EMT in azoxymethane (AOM)/dextran sodium sulfate (DSS)–induced caCRC [16] were evaluated to confirm whether or not SYD can inhibit caCRC through EMT regulation.

## Materials and methods

### SYD preparation

SYD was composed of nine commonly used herbs: Radix Paeoniae Alba, Radix Angelicae Sinensis, Rhizoma Coptidis, Semen Arecae, Radix Aucklandiae, Radix Et Rhizoma Glycyrrhizae, Radix Et Rhizoma Rhei, Radix Scutellariae, Cortex Cinnamomi. The raw herbs for SYD were purchased from the affiliated Nan Fang Hospital of Southern Medical University. These were mixed in the ratio of 30:15:15:6:6:6:9:15:7.5 (dry weight). Aqueous extracts of SYD were extracted at 80°C by stirring for 1 h using 10 volumes of distilled water (v/m). The extracts were centrifuged at 1,500 g at room temperature. To obtain the semisolid SYD solution, the supernatant was collected and subjected to condensation under reduced pressure at 70°C [17]. The quality of SYD was confirmed by HPLC analysis (Additional file 1: Figures S1 and S2, Table S1). SYD was suspended again in 0.9% saline at a final concentration of 2 g/mL. The solution was stored in aliquots at -20°C.

### Animals and experimental procedure

All procedures involving laboratory animal use were in accordance with the guidelines of the Instituted Animal Care and Use Committee of Southern Medical University. All protocols were submitted and validated by Animal Care Ethics Committee of Southern Medical University (No. 2012–055). 6 and 8 weeks male C57BL/6 J mice weighting 15–20 g (specific pathogen-free) were obtained from the Laboratory Animal Center of Southern Medical University. The animals were maintained under controlled conditions (22°C, 12-h/12-h dark/light cycle) in a conventional animal colony. There were 65 mice used in the study, 15 mice in control group, 30 in model group and 20 in SYD group. At the end of the study, there were 15 surviving mice in control group, 7 in model group and 12 in SYD group, respectively.

**Figure 1:**
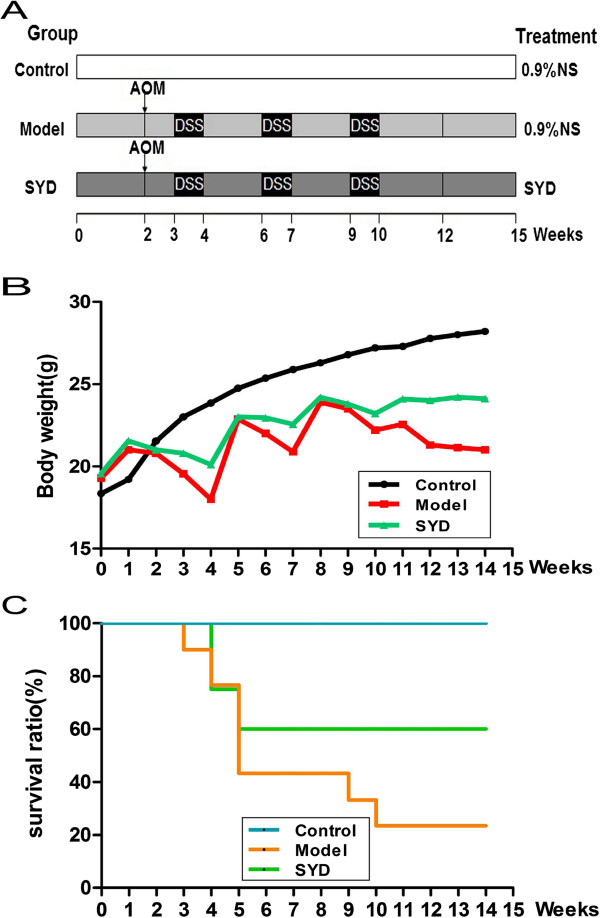
**Effects of SYD on colitis**-**associated colon carcinogenesis was evaluated in C57BL/6 J mice.(A)** Experimental protocol for colitis-associated colon carcinogenesis model. **(B)** Effect of SYD on the body weight of mice. **(C)** Effect of SYD on the survival ratio of mice. SYD prolonged animal survival (P = 0.032, vs. Model). Data are presented as mean ± SD vs. control and model.

Procedures of induction of caCRC model by AOM and DSS was shown in Figure 1A [16]. At week 2, mice were injected with AOM (10 mg/kg, i.p.). After 1 week, 3% DSS (International Lab, Chicago, IL, USA) was added to the drinking water for 7 days followed by 14 days of tap water for recovery. This cycle was repeated twice.

SYD (7.12 g/kg) or equivalent normal saline was administered by gavage using a tube twice a day. The mice were killed by cervical dislocation at week 16 and the colons (from the ileocecal junction to the anal verge) were removed. After measuring the length and weight, the colons were cut longitudinally along the main axis, washed with phosphate-buffered saline (*p*H 7.4, 4°C) and macroscopically inspected. The number, size and location of pre-neoplastic and neoplastic lesions (dysplasia and carcinoma) in the colons were recorded based on gross examination. The size of dysplasia was measured by an ocular micrometer. After gross examination, the colons were cut into pieces at about 1 cm intervals. Samples were then fixed in 10% buffered formalin (*p*H 7.4) or kept in 10 volumes of RNAlater Solution (Ambion, Life technologies, Carlsbad, CA, USA). Remaining samples were flash-frozen in liquid nitrogen and stored at -80°C for further immunohistochemistry, PCR and protein analysis.

### Reagents

Paeonolsilatie sodium was purchased from Jinling Pharmaceutical Co., Ltd. Dimethyl sulfoxide (DMSO) and thiazolyl blue tetrazolium bromide (MTT) were purchased from Sigma-Aldrich (St. Louis, MO, USA). RPMI 1640, FBS and antibiotics were purchased from Invitrogen (Gibco,Grand Island,NY, USA). Snail homolog 1 (Drosophila) was purchased from FulenGen (Genecopoeia,USA).

### Cell viability assays

MTT assay was used to analyze the effects of paeonol and SYD on cell viability in two kinds of colorectal adenocarcinoma cell lines SW480,HCT116. Cells were routinely maintained in RPMI 1640, supplemented with 10% FBS and antibiotics (50 U/ml of penicillin and 50 μg/ml streptomycin) at 37°C in a humidified atmosphere containing 5% CO2. Cell number was measured on a standard microscope by manual counting of cells using a grid with an area of 0.25 mm^2^. 1 × 10^4^ Cells were seeded on 96-well plates in a 100 μl volume. The MTT assay was performed by addition of MTT to a final concentration of 0.5 mg/ml of medium. After 4 h, the medium was removed, and 150 μl of DMSO was added to each well. After 10 min of shaking at room temperature, 150 μl of soluble material was placed into a new 96-well plate, and absorbance at 562 nm was read with a background subtraction at 660 nm.

### Cell culture and transfection

HCT116 cell line was cultured in RPMI 1640, supplemented with 10% FBS and antibiotics (50 U/ml of penicillin and 50 μg/ml streptomycin) at 37°C in a humidified atmosphere containing 5% CO2. For transfection,cells were seeded in a 6-well plate 24 h before the experiment. The cells were transfected with Snail 1 plasmid using the Attractene reagent (Qiagen, Germany) according to the manufacture’s protocol.

### Histopathological assessment

For histopathological examination, formalin fixed, paraffin-embedded colon tissues were cut into serial sections (5 μm) and stained with haematoxylin and eosin (H&E). Histological alternations such as dysplasia and carcinoma were verified. Assessment of dysplasia and cancer was based on the criteria set forth in the Mouse Models of Intestinal Cancers consensus report: location, presence/absence of prolapse/herniation, size, dysplasia grade, invasion level, presence of desmoplasia and presence of irregular architecture [18]. Carcinoma was defined as high-grade dysplasia of colonic mucosa which invaded beyond the muscularis mucosa and into the submucosa [16].

### Immunohistochemistry

Paraffin-embedded colon sections were deparaffinized, rehydrated, and pre-treated with hydrogen peroxidase in PBS buffer. Heat-induced antigen retrieval was performed. After blocking with the appropriate antisera sections were incubated with anti-Proliferating cell nuclear antigen (PCNA) (Thermo scientific, clone PC10, 1:300), anti-β-catenin (Cell Signaling Technology, CST, clone 6B3; 1:100 dilution), anti-p53 (Leica-microsystems, clone CM5, 1:100 dilution), anti-COX-2 (Thermo scientific, 1:100 dilution), anti-E-cadherin ( CST, clone24E10, 1:200 dilution), anti-N-cadherin (Millipore, clone EPR1792Y, 1:50 dilution), anti-fibronectin (Epitomics, clone F14, 1:200 dilution), anti-vimentin (CST, clone D21H3, 1:50 dilution), NF-κB (Abcom, clone E379, 1:100 dilution), F4/80 (Santa Cruz Biotechnology, clone BM8, 1:50 dilution). After incubation with HRP-conjugated secondary antibody and tyramide amplification followed by streptavidin-HRP, positive signals were visualized by DAB kit and counter-stained with hematoxylin. Five randomly selected fields from each section were examined at a magnification of 400× and analyzed using NIS-Elements. The positive content was calculated using the following formula: positive content (PC) = mean optical density × positive area.

### Western blot analysis

Colonic tissues were homogenized in liquid nitrogen, dissolved in lysis buffer [7 mol/L urea, 2 mol/L thiourea, 4% (w/v) CHAPS, 20 mmol/L Tris, 65 mmol/L DTT, 0.2% pharmalyte 3/10 ampholyte] supplemented with 8 μl of protease inhibitor cocktail (The protease inhibitor cocktail include AEBSF hydrochloride 500 μM, Aprotinin 150 nM, E-64 protease inhibitor 1 μM, EDTA disodium 0.5 mM and Leupetin hemisulfate 1 μM. Calbiochem, San Diego, CA, USA). Samples were then centrifuged at 15,000 g for 30 min at 4°C. Protein concentrations were determined by modified Bradford assay and resolved by SDS-PAGE, transferred to PVDF membranes and blocked in 5% non-fat milk in Tris-buffered saline (TBST, 100 mM NaCl, 50 mM Tris, 0.1% Tween-20, pH 7.5). Membranes were incubated overnight with primary antibodies [anti-PCNA (CST, clone PC10, 1:1000 dilution), anti-β-catenin (CST, clone 6B3, 1:1000 dilution), anti-p53 (CST, clone 1C12, 1:1000 dilution), or anti-COX-2 (CST, clone D5H5, 1:1000 dilution), anti-E-cadherin (CST, clone 24E10, 1:1000 dilution), anti-N-cadherin (Millipore, clone EPR1792Y, 1:50000 dilution), anti-Vimentin (CST, clone D21H3, 1:1000 dilution), anti-Snail (CST, clone C15D3, 1:1000 dilution)] at 4°C. This was followed by incubation with horseradish peroxidase (HRP) conjugated secondary antibodies. Protein bandings were visualized with enhanced chemiluminescence reagents (ECL, GE Healthcare Bio-science, Uppsata, Sweden). The images were captured with a CCD system (imagestation 2000 MM, Kodak, Rochester, NY, USA). Quantitative analysis of signals was performed using Molecular Imaging Software Version 4.0, provided by Kodak 2000 MM System. The optical density was normalized by β-actin.

### Cytokine measurement by multiplex immunoassay

The levels of interleukin-1β (IL-1β), IL-6 and TNF-α in the serum were measured using mice cytokine multiplex kit (Millipore, Billerica, MA, USA). Quantification of cytokines was performed using the Luminex system (Austin, TX, USA) [19].

### Statistical analysis

Each experiment was repeated at least three times. Data were presented as mean ± SD. All data were analyzed using SPSS statistical package (version 13.0, SPSS Inc, Chicago, IL, USA). Survival rate was analyzed with Kaplan-Meier survival analysis. Data between two groups were compared with 2-independent samples tests. Ranked data were analyzed by nonparametric test (K Independent Samples Test). Mean values of data from more than 3 groups were analyzed by one-way analysis of variance (ANOVA). A value of *P* < 0.05 was considered as statistically significant.

## Result

### SYD increased the survival rate of mice treated with AOM/DSS

Body weight loss and bloody stools were observed in mice acutely exposed to DSS. These symptoms were relieved during the recovery period (Figure 1B). The colon weight was increased and the length was shortened significantly in the mice that received both AOM and DSS compared with the control mice (Table 1). The significant increases in colon weight and colon length ratio were caused by apparent mucosal thickening. SYD substantially ameliorated the body weight fluctuation and mucosal thickening. The Kaplan–Meier survival curves showed that SYD significantly increased the survival rate of the mice (Figure 1C).

**Table 1:** Colon and spleen assessment in mice

**Group**	**Colon**			**Spleen**	
	**Weight(g)**	**Length(cm)**	**W/L ratio**	**Weight(g)**	**SW/BW ratio**
Control (n = 15)	0.23 ± 0.022	9.14 ± 0.628	0.02 ± 0.003	0.07 ± 0.005	0.003 ± 0.0002
Model (n = 7)	0.54 ± 0.059^△^	5.75 ± 0.477^△^	0.09 ± 0.008^△^	0.35 ± 0.036^△^	0.018 ± 0.0019^△^
SYD (n = 12)	0.37 ± 0.049^*^	7.56 ± 1.133^#^	0.05 ± 0.003^*^	0.18 ± 0.061^*^	0.007 ± 0.0025^*^

*: P < 0.01 VS Model; ^#^: P < 0.05 VS Model.

△: P < 0.01 VS Control.

### SYD reduced the incidence and multiplicity of colonic neoplasms and oncogenic protein expression

**Figure 2:**
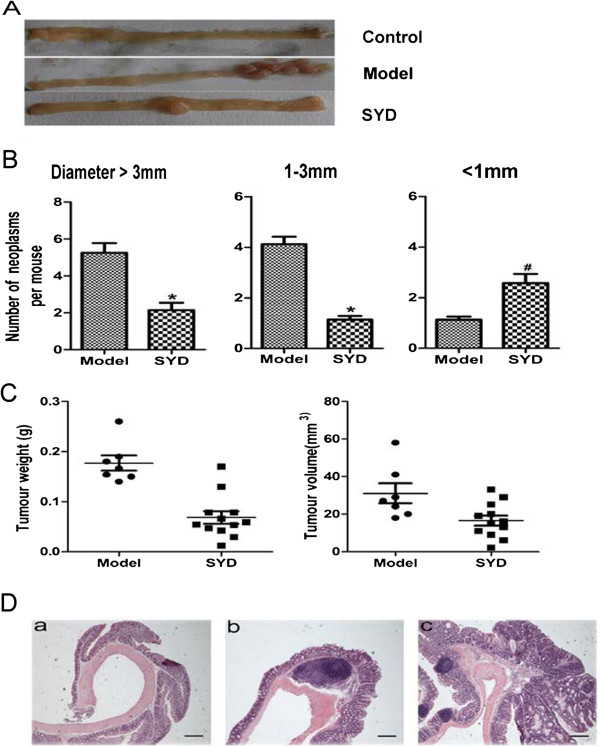
**Effects of SYD on the burden of colonic neoplasms.(A)** Microscopic view of colon in mice. **(B)** Effect of SYD on colonic neoplasm size. SYD significantly suppressed tumor growth and reduced neoplasm size. Data are presented as mean ± SD. *P < 0.01 vs. model, # P < 0.05 vs. model. **(C)** Effects of SYD on tumour weight and tumour volume in each mice. SYD significantly decreased tumour weight and tumour volume. **(D)** Most colorectal neoplasms were histologically consistent with tubular adenoma or adenocarcinoma. Histological studies were carried out through hematoxylin and eosin staining as previously described. (Da) Normal colon; (Db) tubular adenoma; (Dc) adenocarcinoma. 40 × for all, scale bar = 100 μm. The analysis of differentiation and invasion is shown in Table 3 (P = 0.041).

The colonic neoplasm formation rate was 100% in the mice treated with AOM/DSS. Neoplasms were principally distributed in the middle and distal colon (Figure 2A). The total multiplicity of colorectal neoplasms decreased by 42.8% after SYD treatment (P < 0.01, Table 2). Moreover, SYD significantly retarded the development of large neoplasms (diameter > 3 mm) by 59.2% (Figure 2B, Table 2). The tumour weight and tumour volume were decreased by SYD (Figure 2C). The neoplasms in the colon were mostly tubular adenoma or adenocarcinoma according to the criteria of Mouse Models of Intestinal Cancers (Figure 2D). The neoplasms was mostly high-differentiation with invasion in model group.SYD treatment could ameliorate level of differentiation and invasion of neoplasms (P = 0.041, Table 3).

**Table 2:** Number and size of neoplasms in colon in mice

**Group**	**Number of neoplasms**			
	**Total number**	**>3 mm**	**1-3 mm**	**<1 mm**
Control (n = 15)	0.00 ± 0.000	0.00 ± 0.000	0.00 ± 0.000	0.00 ± 0.000
Model (n = 7)	10.25 ± 1.669^△^	5.25 ± 1.488^△^	4.13 ± 0.835^△^	1.13 ± 0.354^△^
SYD (n = 12)	5.86 ± 1.574^*^	2.14 ± 1.069^*^	1.14 ± 0.378^*^	2.57 ± 0.976^#^

*: P < 0.01 VS Model; ^#^: P < 0.05 VS Model.

△: P < 0.01 VS Control.

**Table 3:** Analysis of differentiation and invasion

**Group**	**Differentiation and invasion(number)**			
	**PD**	**MD**	**HD-I**	**HD-NI**
Model (n = 7)	0	0	7	0
SYD (n = 12)	0	1	4	7

PD: poor differentiation; MD: middle differentiation.

HD-I: high differentiation and invasion.

HD-NI: high differentiation and noninvasion.

**Figure 3:**
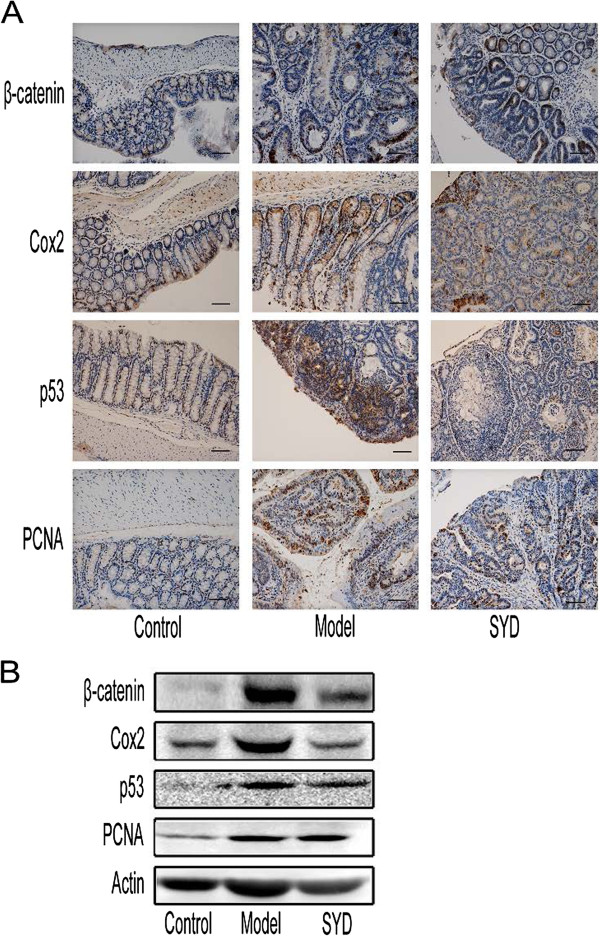
**Effects of SYD on oncogenic protein expression.(A)** Immunohistochemical staining of β-catenin, Cox2, p53, and PCNA in tumor tissues or normal colonic tissues. β-catenin, p53, and PCNA were localized within the nuclei. 200× for all, scale bar = 100 μm. **(B)** Western blot of β-catenin, Cox2, p53, and PCNA expression in colonic tissues. The expression levels of these proteins were reduced in the SYD group compared with the model group. Semiquantitive analysis of these proteins is showed in Table 4.

The expression levels of proliferating cell nuclear antigen (PCNA) and β-catenin increased in dysplastic and neoplastic lesions (Figure 3A and Figure 3B). Intense nuclear staining for p53 (CM5 clone: detects both mutant and wild-type forms) was detected in the neoplastic epithelium and in nondysplastic crypts, implicating the involvement of p53 mutation in our AOM/DSS-induced caCRC model [18]. The expression levels of these neoplastic markers suggested that AOM/DSS-induced caCRC phenotypically resembled human caCRC even though the expression of COX-2 was not high in adenocarcinoma (Figures 3A and 3B, Table 4).

**Table 4:** Semiquantitive analysis of IHC and WB

	**Control**	**Model**	**SYD**
**β**-**catenin**-**IHC**	**0.52** ± **0.39**	**4.12** ± **1.83**^△^	**2.23** ± **1.92**^#^
Cox2-IHC	0.97 ± 0.30	6.18 ± 2.02^△^	3.06 ± 1.53*
p53-IHC	0.66 ± 0.45	6.03 ± 2.17^△^	3.33 ± 2.07^#^
PCNA-IHC	1.26 ± 0.60	5.63 ± 2.21^△^	3.06 ± 1.95^#^
E-cadherin-IHC	0.50 ± 0.39	0.09 ± 0.07	3.44 ± 1.69^#^
N-cadherin-IHC	0.51 ± 0.38	8.10 ± 1.52^△^	4.22 ± 1.19^#^
Fibronectin-IHC	0.80 ± 0.81	7.48 ± 2.08^△^	4.14 ± 1.59^#^
Vimentin-IHC	0.24 ± 0.28	4.92 ± 1.73^△^	2.84 ± 1.46^#^
NF-κB-IHC	0.89 ± 0.48	8.29 ± 1.53^△^	4.69 ± 1.83^#^
F4/80-IHC	0.03 ± 0.04	6.05 ± 1.61^△^	3.89 ± 1.54^#^
β-catenin-WB	0.008 ± 0.006	0.750 ± 0.292^△^	0.250 ± 0.270^#^
Cox2-WB	0.277 ± 0.070	0.677 ± 0.239^▲^	0.307 ± 0.074^#^
p53-WB	0.064 ± 0.040	0.370 ± 0.105^△^	0.165 ± 0.073^#^
PCNA-WB	0.089 ± 0.0226	0.282 ± 0.091^△^	0.158 ± 0.039^#^
E-cadherin-WB	0.723 ± 0.229	0.194 ± 0.099^▲^	0.560 ± 0.185^#^
N-cadherin-WB	0.096 ± 0.055	0.620 ± 0.271^▲^	0.262 ± 0.131^#^
Vimentin-WB	0.217 ± 0.038	0.583 ± 0.200^▲^	0.290 ± 0.110^#^
Snail-WB	0.085 ± 0.037	0.423 ± 0.146^△^	0.187 ± 0.096^#^
Claudin-1-WB	0.018 ± 0.017	0.280 ± 0.141^△^	0.109 ± 0.027^#^

*: P < 0.01 VS Model; ^#^: P < 0.05 VS Model.

△: P < 0.01 VS Control; ^▲^: P < 0.05 VS Control.

IHC: Immunohistochemistry; WB: Western blot.

### SYD and Paeonol inhibited CRC cell proliferation

**Figure 4:**
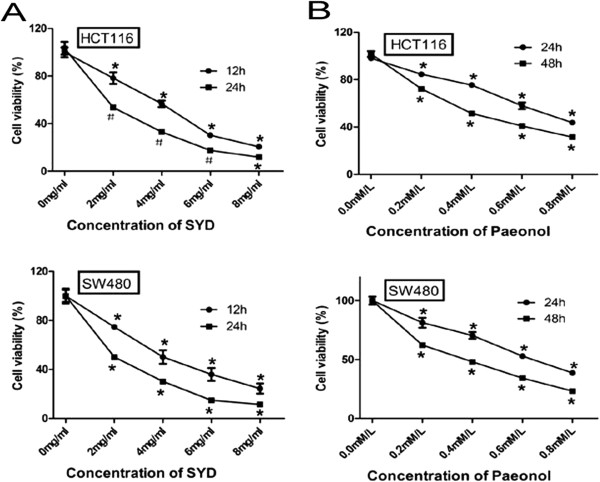
**Effects of SYD and Paeonol on colorectal cancer cells in vitro.(A)** Effects of SYD on proliferation of SW480 and HCT116. Cells were exposed to 0–8 mg/mL SYD and incubated for 12 and 24 h. Cell viability was measured by MTT methods and values were expressed as percentage of the cell viability compared to 0-only. *P < 0.01 vs. 0 mg/mL; #P < 0.05 vs. 0 mg/mL. **(B)** Effects of Paeonol on proliferation of SW480 and HCT116. Cells were exposed to 0–0.8 mM/L Paeonol and incubated for 24 and 48 h. Cell viability was measured by MTT methods and values were expressed as percentage of the cell viability compared to 0-only. *P < 0.01 vs. 0 mM/L; #P < 0.05 vs. 0 mM/L.

SYD significantly decreased cell viability in a time- and dose-dependent manner; the dose ranged from 0 mg/mL to 8 mg/mL. The viabilities of SW480 and HCT116 cells decreased to 50.2266% and 53.6704% after 24 h of treatment with 2 mg/mL SYD (Figure 4A). Paeonol, an ingredient of Radix Paeoniae Alba, significantly decreased cell viability in a time-and dose-dependent manner; the dose ranged from 0 mM/L to 0.8 mM/L. The viabilities of SW480 and HCT116 cells decreased to 48.0659% and 51.3992% after 48 h of treatment with 0.4 mM /L Paeonol (Figure 4B).

### SYD and Paeonol decreased oncogenic proteins in CRC cells

**Figure 5:**
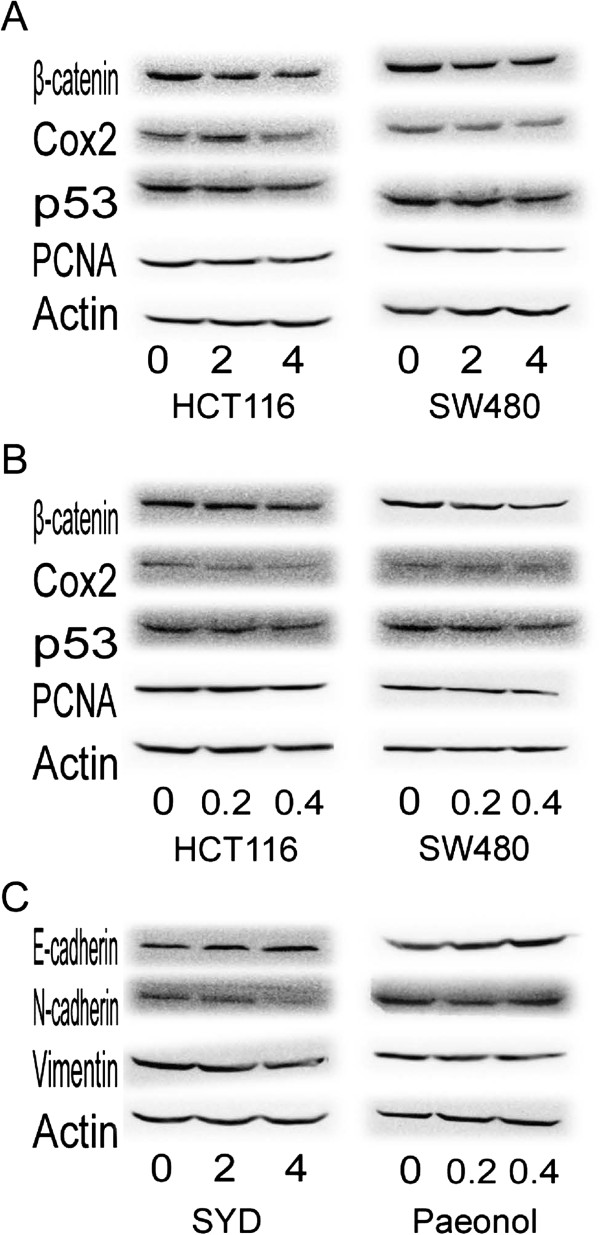
**Effects of SYD and Paeonol of oncogenic protein expression on colorectal cancer cells**, **HCT116,SW480.(A)** Western blot of β-catenin, Cox2, p53 and PCNA expression in SW480 and HCT116 exposed to 0–4 mg/mL SYD. The expression levels of these proteins were reduced in a dose-dependent manner. Semiquantitive analysis of these proteins is showed in Table 5. **(B)** Western blot of β-catenin, Cox2, p53 and PCNA expression in SW480 and HCT116 exposed to 0–0.4 mM/L Paeonol. The expression levels of these proteins were reduced in a dose-dependent manner. Semiquantitive analysis of these proteins is showed in Table 6. **(C)** Western blot of E-cad, N-cad and vimentin expression in SW480 with Snail overexpression exposed to 0–4 mg/mL SYD and 0–0.4 mM/L Paeonol. The expression levels of N-cad and vimentin were reduced in a dose-dependent manner, while E-cad was increased in a dose-dependent manner. Semiquantitive analysis of these proteins is showed in Tables 5 and 6.

SYD and Paeonol decreased the expression levels of PCNA, β-catenin, p53, and COX-2 in CRC cells in a dose-dependent manner. (Figure 5A and Figure 5B, Table 5 and Table 6).

**Table 5:** Semiquantitive analysis of WB of SYD on HCT116 and SW480

	**0 mg/mL**	**2 mg/mL**	**4 mg/mL**
β-catenin-H	0.61 ± 0.03	0.33 ± 0.06*	0.193 ± 0.07*
Cox2-H	0.24 ± 0.03	0.13 ± 0.03*	0.09 ± 0.03*
p53-H	0.52 ± 0.06	0.35 ± 0.06^#^	0.19 ± 0.06*
PCNA-H	0.32 ± 0.06	0.17 ± 0.08^#^	0.11 ± 0.04*
β-catenin-S	0.68 ± 0.009	0.34 ± 0.06*	0.28 ± 0.02*
Cox2-S	0.08 ± 0.01	0.02 ± 0.01*	0.008 ± 0.001*
p53-S	0.55 ± 0.10	0.34 ± 0.08^#^	0.25 ± 0.09*
PCNA-S	0.32 ± 0.12	0.09 ± 0.06^#^	0.06 ± 0.03*
E-cadherin-SS	0.09 ± 0.03	0.30 ± 0.08^#^	0.51 ± 0.12*
N-cadherin-SS	0.22 ± 0.05	0.09 ± 0.02*	0.06 ± 0.02*
Vimentin-SS	0.53 ± 0.06	0.35 ± 0.07^#^	0.15 ±0.07*

*: P < 0.01 VS 0; ^#^: P < 0.05 VS 0; H: HCT116; S: SW480; SS: SW480 with Snail overexpression.

**Table 6:** Semiquantitive analysis of WB of Paeonol on HCT116 and SW480

	**0 mM/L**	**0.2 mM/L**	**0.4 mM/L**
β-catenin-H	0.38 ± 0.04	0.22 ± 0.03*	0.13 ± 0.04*
Cox2-H	0.17 ± 0.07	0.04 ± 0.008^#^	0.01 ± 0.002*
p53-H	0.35 ± 0.14	0.17 ± 0.07^#^	0.08 ± 0.04*
PCNA-H	0.37 ± 0.09	0.19 ± 0.08^#^	0.09 ± 0.04*
β-catenin-S	0.21 ± 0.03	0.13 ± 0.04^#^	0.05 ± 0.03*
Cox2-S	0.14 ± 0.04	0.05 ± 0.01*	0.008 ± 0.002*
p53-S	0.48 ± 0.18	0.20 ± 0.08^#^	0.10 ± 0.05*
PCNA-S	0.22 ± 0.04	0.12 ± 0.04^#^	0.08 ± 0.06^#^
E-cadherin-SS	0.04 ± 0.03	0.23 ± 0.06*	0.35 ± 0.08*
N-cadherin-SS	0.42 ± 0.06	0.30 ± 0.08^#^	0.15 ± 0.03*
Vimentin-SS	0.37 ± 0.05	0.18 ± 0.05*	0.07 ± 0.04*

*: P < 0.01 VS 0; ^#^: P < 0.05 VS 0; H: HCT116; S: SW480; SS: SW480 with Snail overexpression.

### SYD inhibited Snail-induced EMT

**Figure 6:**
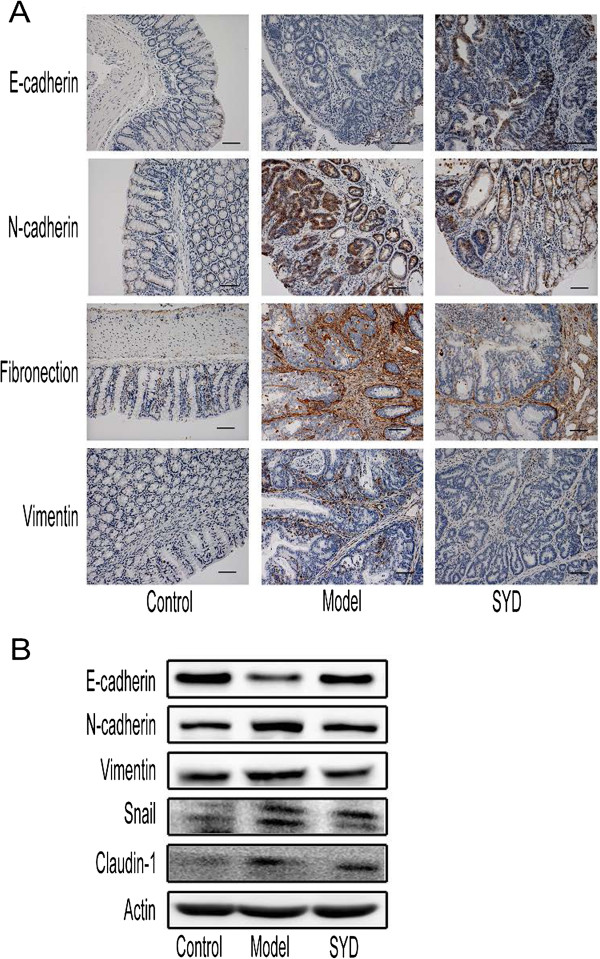
**Effects of SYD on EMT**-**related protein expression.(A)** Immunohistochemical staining of E-cadherin, N-cadherin, fibronectin, vimentin in tumor tissues or normal colonic tissues. 200 × for all, scale bar = 100 μm. **(B)** Western blot of E-cadherin, N-cadherin, vimentin, Snail, and claudin-1 in colonic tissues. E-cadherin was significantly deficient in the model group. E-cadherin was upregulated while N-cadherin, fibronectin, and vimentin were downregulated by SYD treatment. These effects were confirmed by Western blot. Semiquantitive analysis of these proteins is showed in Table 4.

E-cad was moderately expressed in the epithelial cells of the control group but was downregulated in the epithelial cells of the model group. N-cadherin (N-cad) and fibronectin were strongly expressed and vimentin was moderately expressed in the model group, suggesting the occurrence of EMT (Figures 6A). SYD significantly increased the expression of E-cad and decreased the expression of N-cad, fibronectin, and vimentin (Figures 6A, Table 4). Western blot analysis further confirmed the effects of SYD on the expression of E-cad, N-cad, and vimentin. Snail was upregulated in the model group, and it was downregulated by SYD (Figure 6B, Table 4).

Overexpression of Snail, a transcription factor crucial in EMT, induced the upregulation of N-cad and vimentin. SYD and Paeonol decreased the expression of N-cad induced by Snail overexpression. (Figure 5C, Table 5 and Table 6)

### SYD decreased serum cytokines, macrophage proliferation, and NF-κB expression

**Figure 7:**
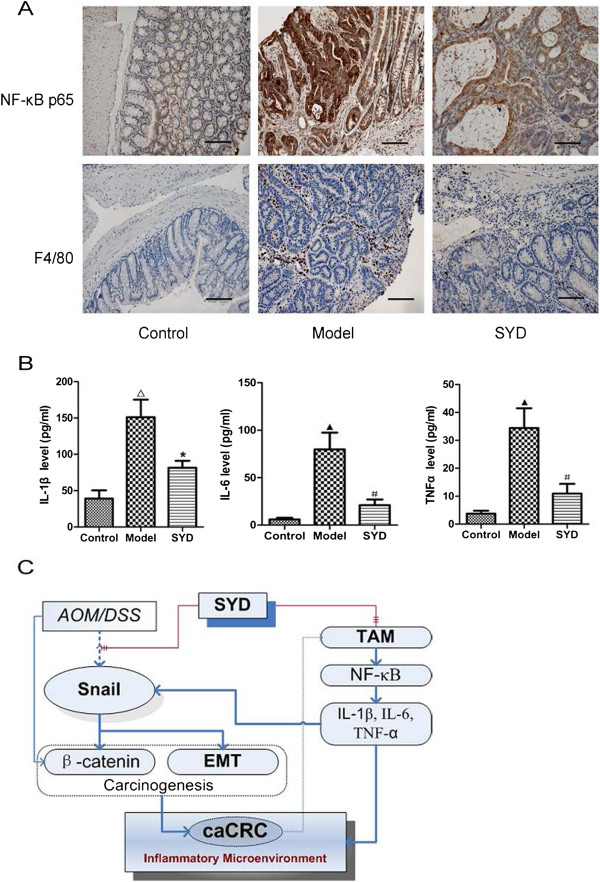
**Effects of SYD on TAM and cytokine profile.(A)** Immunohistochemical staining of NF-κB and F4/80 in tumor tissues or normal colonic tissues .200 × for all, scale bar = 100 μm. High expression levels of NF-κB and F4/80 were detected in the model group. SYD decreased NF-κB expression and F4/80-positive cells. Semiquantitative analysis of these proteins is shown in Table 4. **(B)** Luminex assay of IL-1β, IL-6, and TNF-α levels in serum. Serum contents of IL-1β, IL-6, and TNF-α were reduced by SYD. Data are presented as mean ± SD. *P < 0.01 vs. model, #P < 0.05 vs. model; △P < 0.01 vs. control, ▲P < 0.0 5 vs. control. **(C)** A model of SYD function in caCRC.

The number of macrophages and the expression of NF-κB were increased significantly in the model group compared with those in the control group; SYD counteracted these effects (Figure 7A). Serum IL-1β, IL-6, and TNF-α were upregulated in the model group and were significantly inhibited by SYD (Figure 7B).

## Discussion

Variations in climatic conditions, geographic locations, and harvesting, processing, and extraction methods could challenge the quality control of herbal extracts from Chinese compound prescriptions [20]. In the present study, several specific chemical markers and batch-to-batch comparison for quality controls were utilized. This strategy (i.e., formulomics) was based on liquid chromatography for chemical characterization and chemical fingerprinting of SYD [21,22]. The strict extract quality control of SYD is important to translate the TCM formula to clinical drugs [21].

Basing on the capacity of SYD to downregulate the expression of proinflammatory cytokines, such as ICAM-1 and TNF-α, we hypothesized that SYD ameliorates caCRC by suppressing inflammation [23,24]. Repeated DSS administration causes chronic inflammation, and AOM induces the formation of O6-methylguanine upon metallic activation [25]. Therefore, DSS in combination with AOM resulted in 100% incidence of colonic neoplasms in mice in the present experiment. DSS administration caused mass death in the model group; the effect of this drug was alleviated by SYD treatment. Tumor numbers were reduced and CRC cell proliferation was inhibited by SYD. These results suggested that SYD elicited antitumor effects by inhibiting CRC cell proliferation.

Increased PCNA and β-catenin expression suggested that DSS/AOM-induced neoplasm resembled human caCRC. The increase in β-catenin due to the constitutive Wnt activation for AOM induces mutations in the exon 3 of Ctnnb [25]. Wild-type p53 is a rapidly degraded protein with a low cellular level [26]; thus, the accumulation of p53 in the model group also characterizes caCRC for the early loss of p53 function [18]. Downregulation of PCNA, β-catenin, and p53 by SYD further confirmed the efficacy of SYD in DSS/AOM-induced caCRC.

EMT disrupts cell–cell adherence, causes apico-basal polarity loss, and triggers matrix remodeling to promote CRC pathogenesis and metastasis [27]. EMT endows cells with migratory and invasive capacity, induces stem cell properties, prevents apoptosis and senescence, and contributes to immunosuppression [12]. In the present study, SYD ameliorated DSS/AOM-induced EMT, as indicated by upregulated E-cad and downregulated N-cad, fibronectin, and vimentin. In a previous report, Snail overexpression results in dramatic changes in signaling pathways that are involved in EMT and contributes to the acquisition of stem/progenitor-like character [28]. Snail also represses epithelial gene expression and thereby promotes EMT and tumor invasion by activating the beta-catenin pathway [29]. SYD and paeonol significantly decreased Snail-induced expression of N-Cad and vimentin. This result suggested that SYD executed its protective activity by downregulating Snail-induced EMT [28,29].

The major function of SYD was to resolve colorectal inflammation through heat clearing and detoxification. Paeonol decreases lipopolysaccharide-induced TNF-α, IL-1β, and IL-6 in RAW 264.7 cells by deactivating IκBα [30]. In addition, the levels of TNF-α, IL-1β, and IL-6 are significantly higher in CRC patients than in controls [31]. In the present study, SYD decreased the serum levels of IL-1β, IL-6, and TNF-α. Reduced IL-1β from macrophages may attenuate the stabilization of β-catenin and deactivate Wnt target genes in CRC cells [32]. Repressed TNF-α and IL-6 inhibit tumor growth in experimental models of colitis-associated cancer [33]. In the present study, macrophage infiltration and NF-κB expression were decreased. IL-1β, IL-6, and TNF-α are multifunctional NF-κB-regulated cytokines that feature κB or κB-like binding motifs in their 5′ regulatory region [34,35]. Thus, we supposed that SYD can attenuate caCRC by suppressing NF-κB activation and related inflammatory responses [36].

Mutant p53 accumulated in inflamed colon and in cancerous glands concomitantly with NF-κB activation and sustained DNA damage. Mice were susceptible to chronic inflammation. However, it also greatly accelerated the inflammation-associated colon cancer under this situation [37]. TNF-α is a critical factor produced by macrophages to accelerate EMT [38]. This factor increases Snail protein expression and Snail nuclear translocation to initiate cancer invasion and metastasis through EMT activation [27]. EMT can be induced by activating the NF-κB pathway and enhancing Snail [39]. AOM exacerbated DSS-induced colitis to caCRC by triggering p53 mutation, β-catenin accumulation, and EMT induction upon tumor-associated macrophage (TAM) infiltration, and NF-κB promoted IL-1β, IL-6, and TNF-α release. Therefore, SYD might ameliorate caCRC by suppressing NF-κB activation and cytokine-induced EMT.

## Conclusions

SYD repressed IL-1β, IL-6, and TNF-α, thereby inhibiting Snail-induced EMT by attenuating TAM infiltration and NF-κB activation [40-42] (Figure 7C).

## Competing interests

The authors declare that they have no competing interests.

## Authors’ contributions

XS and XY designed the study. XL, JD, and MS carried out experiments. ZY and QF performed statistical analysis. LZ, HC participated in the design of study, interpretation of results. All authors read and approved the final manuscript.

## Supplementary Material

Additional file 1: Figure S1 Similarity analyses of chromatographic SYD samples. The similarities of repeatability of 6 batches of SYD were from 0.937 to 0.9776. **Table S1.** Comparability result of reproducibility of SYD samples. **Figure S2.** Determination of berberin and baicalein in SYD sample. Berberin and baicalein were matched in SYD sample. Click here for file
