# Supplementary material for: ShaoYao decoction ameliorates colitis-associated colorectal cancer by downregulating proinflammatory cytokines and promoting epithelial-mesenchymal transition
Source: J Transl Med. 2014 Apr 26;12:105. doi: 10.1186/1479-5876-12-105 (PMC4022058; doi:10.1186/1479-5876-12-105)
Supplement: Additional file 1: Figure S1 — Similarity analyses of chromatographic SYD samples. The similarities of repeatability of 6 batches of SYD were from 0.937 to 0.9776. Table S1. Comparability result of reproducibility of SYD samples. Figure S2. Determination of berberin and baicalein in SYD sample. Berberin and baicalein were matched in SYD sample. [file 1479-5876-12-105-S1.doc]

**Supplementary material**

**Compositional analysis of SYD by HPLC**

Instruments: High performance liquid chromatography (HPLC) analysis of SYD was performed on Shimadzu HPLC system (Shimadzu Co., Kyoto, Japan) using a C_18_ column Shimadzu VP ODS (250×4.6 mm; particle size 5 μm, Japan).

Method: The mobile phases comprised eluent A (0.5 ‰ aqueous acetic acid) and eluent B (acetonitrile). The gradient flow was as follows: 0.00–80.00 min, 10%–50% B; 80.01–95.00 min, 95% B; 95.01–100.00 min, 50% B; 100.01–110.00 min, 10% B. The analysis was performed at a flow rate of 1.0 mL/min with PDA detection at 254 nm. The injection volume was 25 μL.

**The reproducibility of SYD samples:** The Similarity Evaluation System for Chromatographic Fingerprint of TCM (2004 A edition) was used to evaluate the similarities of the 6 batches of SYD. After peak-picking, template-matching process, the peaks in the spectra were matched automatically (Figure S1). The reference template was set finally for spectra peak difference and entire similarity evaluation. The similarities of repeatability were from 0.937 to 0.977 (Table S1)．The results showed that the preparation process of Shaoyao decoction (SYD) was reasonable.

**Determination of berberin and baicalein in SYD sample:** berberin and baicalein was the two main active compounds of Coptis chinensis and Scutellaria baicalensis respectively. The determination of the two compounds by the same HPLC eluted system could cotroll the quality of SYD (Figure S2).





Figure S1 Similarity analyses of chromatographic SYD samples

Table S1. Comparability result of reproducibility of SYD samples

|  | Sample 1 | Sample 2 | Sample 3 | Sample 4 | Sample 5 | Sample 6 | Reference Fingerprint |
| --- | --- | --- | --- | --- | --- | --- | --- |
| Sample 1 | 1.000 | 0.952 | 0.953 | 0.847 | 0.848 | 0.849 | 0.937 |
| Sample 2 | 0.952 | 1.000 | 0.998 | 0.908 | 0.910 | 0.908 | 0.977 |
| Sample 3 | 0.953 | 0.998 | 1.000 | 0.904 | 0.906 | 0.905 | 0.976 |
| Sample 4 | 0.847 | 0.908 | 0.904 | 1.000 | 0.999 | 0.997 | 0.973 |
| Sample 5 | 0.848 | 0.910 | 0.906 | 0.999 | 1.000 | 0.997 | 0.974 |
| Sample 6 | 0.849 | 0.908 | 0.905 | 0.997 | 0.997 | 1.000 | 0.973 |
| Reference Fingerprint | 0.937 | 0.977 | 0.976 | 0.973 | 0.974 | 0.973 | 1.000 |


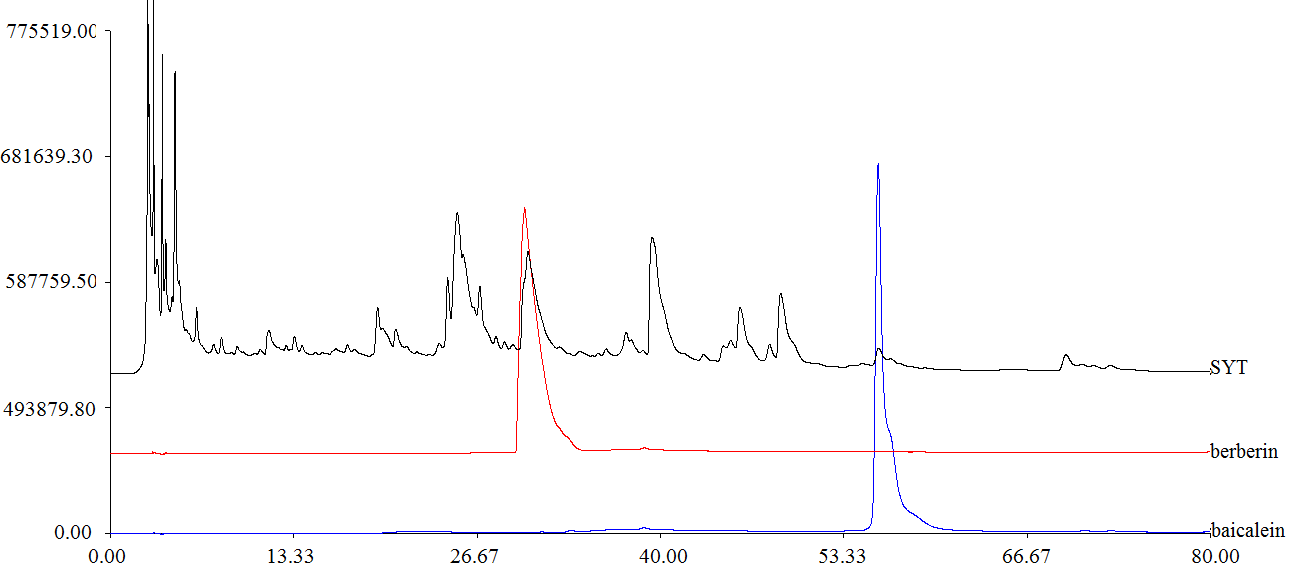


Figure S2 The matched berberin and baicalein in SYD sample
